# Is there a role for the quantification of RRM1 and ERCC1 expression in pancreatic ductal adenocarcinoma?

**DOI:** 10.1186/1471-2407-12-104

**Published:** 2012-03-22

**Authors:** Matias E Valsecchi, Thomas Holdbrook, Benjamin E Leiby, Edward Pequignot, Susan J Littman, Charles J Yeo, Jonathan R Brody, Agnieszka K Witkiewicz

**Affiliations:** 1 Department of Medical Oncology, Thomas Jefferson University, 834 Chestnut Street Suite 320, Philadelphia, PA 19107, USA; 2 Department of Pathology, Thomas Jefferson University and the Jefferson Pancreas, Biliary and Related Cancer Center, Philadelphia, PA, USA; 3 Division of Biostatistics, Department of Pharmacology and Experimental Therapeutics, Thomas Jefferson University, Philadelphia, PA, USA; 4 Department of Surgery, Thomas Jefferson University, Philadelphia, PA, USA; 5 Departments of Surgery and Pathology, Thomas Jefferson University, Philadelphia, PA, USA

## Abstract

**Background:**

RRM1 and ERCC1 overexpression has been extensively investigated as potential predictive markers of tumor sensitivity to conventional chemotherapy agents, most thoroughly in lung cancer. However, data in pancreatic cancer are scarce.

**Methods:**

We investigated the mRNA and protein expression of ERCC1 and RRM1 by RT-PCR and immunohistochemistry (IHC) in formalin-fixed, paraffin-embedded pancreatic ductal carcinoma (PDA) tissues. The primary outcome investigated was the association between RRM1 and ERCC1 expression and overall survival (OS) or disease-free survival (DFS).

**Results:**

A total of 94 patients with resected PDA were included in this study. Most of them (87%) received gemcitabine based chemotherapy. Data for OS analysis was available in all cases but only 68% had enough information to estimate DFS. IHC analysis revealed information for 99% (93/94) and 100% of the cases for RRM1 and ERCC1 expression respectively. However, PCR data interpretation was possible in only 49 (52%) and 79 (84%) cases respectively. There was no significant association between high or low expression of either RRM1 or ERCC1, detected by IHC and OS (14.4 vs. 19.9 months; *P *= 0.5 and 17.1 vs. 19.9; *P *= 0.83 respectively) or PCR and OS (48.0 vs. 24.1 months; *P *= 0.21 and 22.0 vs. 16.0 months; *P *= 0.39 respectively). Similar results were obtained for DFS.

**Conclusions:**

RRM1 and ERCC1 expression does not seem to have a clear predictive or prognostic value in pancreatic cancer. Our data raise some questions regarding the real clinical and practical significance of analyzing these molecules as predictors of outcomes.

## Background

Pancreatic ductal adenocarcinoma (PDA) is recognized as the fourth leading cause of cancer-related mortality, being responsible for almost 40,000 deaths per year in the US [1]. Only 20% of the patients undergo surgical resection and, with the exception of extremely rare circumstances, almost all patients receive some sort of chemotherapy either as neoadjuvant, adjuvant or systemic treatment of metastatic disease. For years, the traditional approach included the use gemcitabine [2] or gemcitabine based combinations [3,4] as the standard of care. This paradigm was recently challenged by the confirmation that another regimen, which includes a combination of 5-FU, leucovorin, oxaliplatin and irinotecan (FOLFIRINOX) was superior to single agent gemcitabine in the metastatic setting [5]. However this benefit was also accompanied by a significant increase in grade 3-4 toxicity. This generates a practical clinical dilemma, especially in those patients who have poor performance status and may not be able to tolerate this regimen. In that sense, information regarding the tumor sensitivity to gemcitabine - and to oxaliplatin - may have useful clinically practical implications [6]. The ribonucleotide reductase subunit M1 (RRM1) and the excision repair cross complementary 1 (ERCC1) enzymes are two of the many proteins that physiologically participate in the synthesis and damage repair of human DNA. Both molecules have been extensively investigated as potential predictive markers of tumor sensitivity to conventional chemotherapy agents. RRM1 is directly affected by gemcitabine, constituting one of its molecular targets [7]. RRM1 inhibition translates into reduced activity of the ribonucleotide reductase complex resulting in decreased production of deoxyribonucleotides needed for the DNA synthesis [8]. It is consequently easy to understand that over-expression of RRM1 can result in gemcitabine resistance [9]. ERCC1, on the other hand, seems to play a more relevant role in the repair of DNA damage resulting from intra and interstrand cross links [10]. Platinum analogues, as a group, exert much of their therapeutic effects through the induction of DNA adducts and cross-links [11]. Consequently, over-expression of ERCC1 and other enzymes able to remove those DNA adducts can translate into tumoral resistance to platinum analogues [12,13]. These two phenomenon have been consistently found to be true especially in non-small cell lung cancer (NSCLC) [14-19]; however it is unclear whether the same concept can be applied to other cancer types, such as PDA. Moreover, it is also uncertain whether high levels of expression of RRM1 and ERCC1 carry any prognostic significance independently of the type of chemotherapy used. At least one previous study showed a better overall survival associated with high levels of RRM1 and ERCC1 in resected PDA [20]. However, this work was never validated in an independent cohort and the sample size was small.

We designed the present clinical study with the objective to determine whether quantification of RRM1 and ERCC1 by immunohistochemical (IHC) and quantitative-PCR analysis has any prognostic or predictive significance in PDA.

## Methods

### Patient selection and data collection

We studied 94 patients with confirmed PDA who underwent surgical resection at The Thomas Jefferson University Hospital between 2002 and 2010, and for whom sufficient material was available for immunohistochemical (IHC) and polymerase chain reaction (PCR) analysis. All had consented to analyses of their tumors via a protocol approved by the Thomas Jefferson University Institutional Review Board. We reviewed the medical charts and contacted primary oncologists to obtain relevant clinical information. Vital status was obtained from medical records and verified by querying the Social Security death index. The following variables were obtained for analysis: age, gender, tumor size and grade, number of lymph nodes resected, number of lymph nodes metastases, resection margin involvement by tumoral cells, type of treatment received (type of surgery, radiation therapy and chemotherapy), time and site of first recurrence and death.

To ensure accuracy, dual data extraction was conducted. Data were subsequently verified between the reviewers and discrepancies resolved through consensus discussion. To minimize subjective judgment and selection bias, reviewers were blinded to clinical outcomes.

### Immunohistochemical and PCR analysis

We investigated the mRNA and protein expression of ERCC1 and RRM1 by RT-qPCR and immunohistochemistry (IHC) in formalin-fixed, paraffin- embedded PDA tissues. Samples were sent by the investigators to Response Genetics (Los Angeles, CA). Relative gene expression quantification was calculated according to the comparative cycle threshold (Ct) method using ß-actin as an endogenous control and commercial RNA controls (Stratagene, La Jolla, CA) as calibrators. Samples were classified as "Low" or "High" expression according to cut off values pre-established by Response Genetics (Los Angeles, CA) [21]. The pancreatic cancer cases originally tested by Response Genetic to establish those cuts off values were not included in the study.

**Figure 1:**
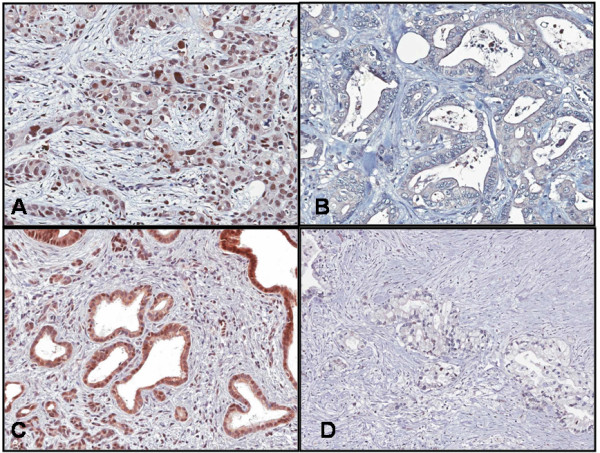
**Staining for RRM1 and ERCC1 proteins**. (**A**) ERCC1-positive sample. Note the intense nuclear staining. (**B**) ERCC1-negative sample. (**C**) RRM1-positive sample. Note the intense cytoplasmic staining. (**D**) RRM1-negative sample.

IHC was performed using purified RRM1 antibody (1:150 dilution; Abcam, Cambridge, MA) and ERCC1 antibody (1:50 dilution; Abcam, Cambridge, MA). RRM1 and ERCC1 immunoreactivity was evaluated semi-quantitatively based on staining intensity and proportion of staining in five representative fields at 400 × magnification. ERCC1 was evaluated based on nuclear staining and RRM1 based on cytoplasmic staining (Figure 1). The stained tumor tissues were scored blindly with respect to clinical patient data. For RRM1, the proportion of staining was scored on a scale from 0 to 3 as follows: > = 50% positive (score 3); 10-49% positive (score 2); 1-9% positive (score 1); negative (score 0). The intensity of staining was scored from 0 to 3 as follows: 0 (absent), 1 (weak), 2 (moderate), 3 (intense). For ERCC1, the proportion of staining was scored on a scale from 0 to 4 as follows: > 50% positive (score 4); 25-50% positive (score 3); 10-24% positive (score 2); 1-9% positive (score 1); negative (score 0). The intensity of staining was scored from 0 to 3 as follows: 0 (absent), 1 (weak), 2 (moderate), 3 (intense) [12]. The immunoreactive score for each case was determined by multiplying the proportion and intensity scores. Criteria for positive staining were modeled after previous work, requiring immunoreactive scores of 9 out of 9 and 6 out of 12 to be considered positive - or high expression - for RRM1 and ERCC1, respectively [22,23].

### Outcomes and statistical analysis

The primary outcome was the association between RRM1 and ERCC1 expression - dichotomized as high or low - and overall survival (OS) or disease-free survival (DFS). OS was defined from the day of surgery to the day of death or last follow-up. DFS was defined from the day of surgery to the day of the first documented relapse or death or last follow -up. Secondary outcomes include the correlation between the IHC and PCR results found for RRM1 and ERCC1 expression.

Patient characteristics were summarized using medians and ranges for continuous variables and frequencies and percentages for categorical variables. The distribution of OS and DFS times was estimated using the Kaplan-Meier method. The log-rank test was used to identify patient and tumor characteristics significantly associated with OS and DFS. Chi-square tests were used to identify covariates associated with RRM1 and ERCC1 expression. Cox proportional hazards regression was used to test for association of RRM1 and ERCC1 expression with OS after adjustment for potential confounders. Spearman rank correlations were calculated to test for association among RRM1 and ERCC1 gene expression levels and IHC scores.

## Results

A total of 94 patients were included in this study, with a median age of 65 years (range: 35-89 years). The median follow up was 15 months. The baseline clinic-pathologic characteristics of the patients are summarized in Table 1. From the whole cohort, 87.2% (82 cases) received gemcitabine based chemotherapy and in 12 cases chemotherapy was not administered. Data for OS analysis were available in all cases but only 64 patients (68%) had enough information to estimate DFS. IHC analysis revealed information for 99% (93/94) and 100% of the cases for RRM1 and ERCC1 expression, respectively. However, PCR data interpretation was possible only in 49 (52%) and 79 (84%) cases respectively.

**Table 1:** Clinico-pathologic characteristics of the 94 patients

Characteristics	N° of Patients (%)	Median (range)
Age (years)	94	66 (35-89)

Gender		
Male	53 (56%)	
Female	41 (44%)	

Size, cm		3 (0-9.5)

Histological Grade		
1	5 (5%)	
2	69 (73%)	
3	18 (19%)	
4	1 (1%)	
(Unknown)^¥^	1 (1%)	

Tumor Size (T)		
1-2	15 (16%)	
3-4	78 (83%)	
(Unknown) ^¥^	1 (1%)	

Nodes (N)		
Positive	65 (69%)	
Negative	27 (29%)	
(Unknown) ^¥^	2 (2%)	

TNM Stage		
IA	2 (2%)	
IB	3 (3%)	
IIA	21 (22%)	
IIB	63 (67%)	
III	3 (3%)	
IV	2(2%)	

Resection Margins		
R0	66 (70%)	
R1	27 (29%)	
(Unknown) ^¥^	1 (1%)	

Vital status		
Alive	34 (36%)	
Dead	60 (64%)	

^¥ ^This corresponds with patients with metastatic disease for whom this variable could not be obtained

There was no significant difference between high or low expression of either RRM1 or ERCC1, detected by IHC or PCR, and any of the clinic-pathological variables analyzed, which included age, race, gender, tumor size, pathological depth (pT), histological grade and presence of metastatic lymph nodes (Table 2).

**Table 2:** Covariates and RRM1 or ERCC1 (Patient N, row%)

	RRM1 Expression								ERCC1 Expression							
	
	RRM1 mRNA expression (PCR)				RRM1 protein expression (IHC)				ERCC1 mRNA expression (PCR)				ERCC1 protein expression (IHC)			
	
Covariate	High	Low	N	*p*	High	Low	N	*p*	High	Low	N	*p*	High	Low	N	*p*
Age (years)																
Young (<65.5)	4 (21%)	15 (79%)	49	1.0	14 (30%)	32 (70%)	93	0.51	19 (48%)	21 (53%)	79	0.36	29 (62%)	18 (38%)	94	0.41
Old (> 65.5)	6 (20%)	24 (80%)			18 (38%)	29 (62%)			14 (36%)	25 (64%)			24 (51%)	23 (49%)		

Size (cm)																
Small (<2.0)	1 (9%)	10 (91%)	49	0.42	5 (26%)	14 (74%)	93	0.59	4 (31%)	9 (69%)	79	0.54	11 (55%)	9 (45%)	94	1.0
Large (> 2.0)	9 (24%)	29 (76%)			27 (36%)	47 (64%)			29 (44%)	37 (56%)			42 (57%)	32 (43%)		

Gender																
Female	6 (24%)	19 (76%)	49	0.73	14 (35%)	26 (65%)	93	1.0	13 (37%)	22 (63%)	79	0.50	25 (61%)	16 (39%)	94	0.53
Male	4 (17%)	20 (83%)			18 (34%)	35 (66%)			20 (45%)	24 (55%)			28 (53%)	25 (47%)		

Race																
White	4 (12%)	29 (88%)	48	0.11	23 (35%)	43 (65%)	92	1.0	22 (42%)	31 (58%)	78	1.0	36 (55%)	30 (45%)	93	0.82
Non-White	5 (33%)	10 (67%)			9 (35%)	17 (65%)			11 (44%)	14 (56%)			16 (59%)	11 (41%)		

Histological Grade^¥^																
1 or 2	7 (19%)	29 (81%)	48	1.0	22 (30%)	51 (70%)	92	0.10	27 (45%)	33 (55%)	78	0.43	42 (57%)	32 (43%)	93	0.80
3 or 4	2 (17%)	10 (83%)			10 (53%)	9 (47%)			6 (33%)	12 (67%)			10 (53%)	9 (47%)		

Lymph Nodes^¥^																
No LN positive	4 (31%)	9 (69%)	47	0.24	9 (35%)	17 (65%)	91	1.0	14 (64%)	8 (36%)	77	0.024	16 (59%)	11 (41%)	92	0.82
At least 1 LN positive	5 (15%)	29 (85%)			23 (35%)	42 (65%)			19 (35%)	36 (65%)			36 (55%)	29 (45%)		

Depth of Tumor (pT) ^¥^																
T1 and T2	2 (20%)	8 (80%)	48	1.0	4 (27%)	11 (73%)	92	0.56	4 (36%)	7 (64%)	78	0.75	5 (33%)	10 (67%)	93	0.087
T3 and T4	7 (18%)	31 (82%)			28 (36%)	49 (64%)			29 (43%)	38 (57%)			47 (60%)	31 (40%)		

^¥ ^The difference in values corresponds to the patients with metastatic disease only, for whom some variables were not able to be computed.

**Figure 2:**
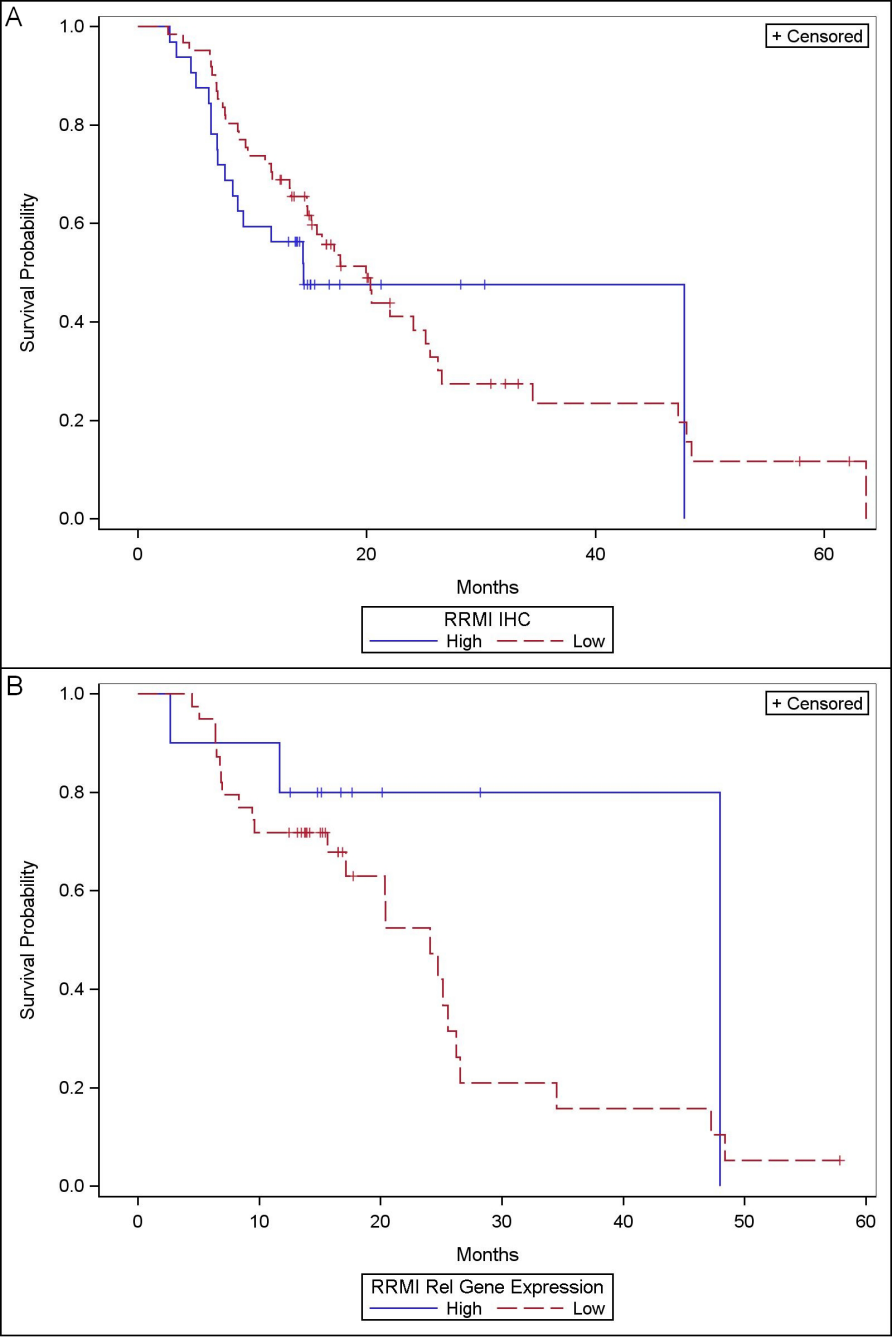
**Kaplan Meier curve for overall survival according RRM1 expression by: **A**) - IHC (*P *= 0.5) and **B**) - PCR (*P *= 0.21)**.

**Figure 3:**
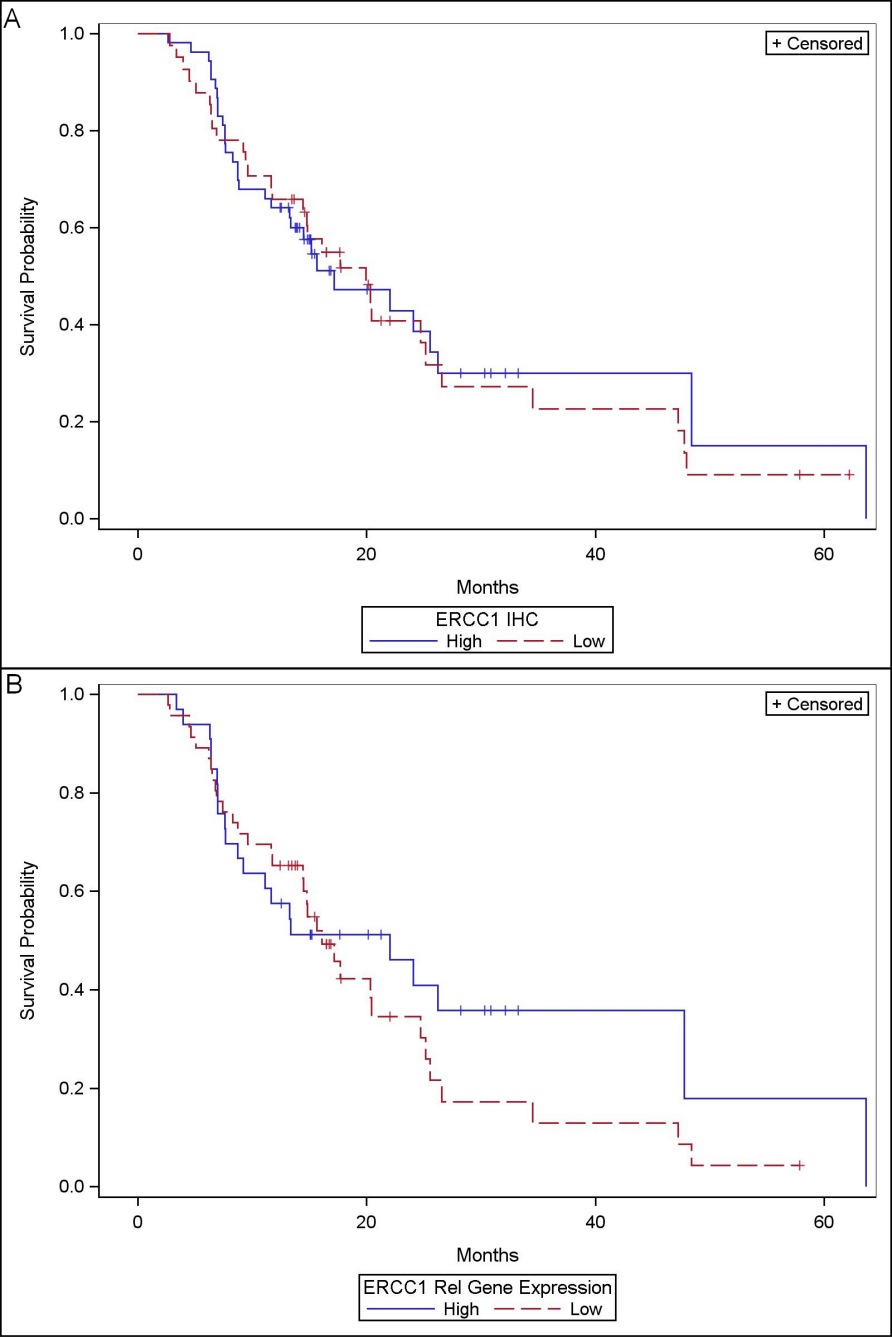
**Kaplan Meier curve for overall survival according ERCC1 expression by: **A**) - IHC (*P *= 0.83) and **B**) - PCR (*P *= 0.39)**.

The median survival of the entire cohort was 18 months (CI: 14-25). In the univariate analysis the variables associated with better OS were younger age (< 65.5 years; *P *= 0.087), histological grade 1 or 2 (*P *= 0.003), absence of lymph node metastases (*P *= 0.009) and use of chemotherapy (*P *< 0.001). However, there was no significant association between high or low expression of either RRM1 or ERCC1, detected by IHC and OS (14.4 vs. 19.9 months; *P *= 0.5 and 17.1 vs. 19.9; *P *= 0.83 respectively) or PCR and OS (48.0 vs. 24.1 months; *P *= 0.21 and 22.0 vs. 16.0 months; *P *= 0.39 respectively) (Figures 2 and 3). Similar results were obtained with DFS where only histological grade 1 or 2 (*P *= 0.031), absence of lymph node metastases (*P *= 0.08) and use of chemotherapy (*P *= 0.013) showed significant longer DFS. The same as in OS, the expression levels of RRM1 or ERCC1 detected by IHC or by PCR did not showed any statistically significant result (Table 3). Using Cox regression multivariate analysis the expression levels of RRM1 and ERCC1 were not significant associated with any change in the OS or the DFS using IHC (Table 4). RRM1 was associated with OS using PCR after adjustment for other predictors of survival. The exclusion of the 12 patients who did not receive a full course of chemotherapy did not make any significant change in the estimates (Table 5).

**Table 3:** Univariate Overall and Disease-Free Survival: Kaplan-Meier medians and log-rank test

		Overall Survival				Disease-Free Survival			
**Covariate**		**Median [months] (95% CI)**	**N**	**Total N**	**Log-rank *p*-value**	**Median [months] (95% CI)**	**N**	**Total N**	**Log-rank *p*-value**

**Overall**		18 (14, 25)	94	94		12 (8, 19)	64	64	

**Age (year)**	**Young (≤ 65.5)**	20 (16, 47)	47	94	0.087	11 (8, 19)	38	64	0.71
	**Old (> 65.5)**	15 (9, 24)	47			14 (7, 25)	26		

**Size (cm)**	**Small (≤ 2.0)**	25 (9, 47)	20	94	0.60	21 (8, ∞)	12	64	0.17
	**Large (> 2.0)**	17 (13, 22)	74			11 (7, 17)	52		

**Gender**	**Female**	20 (12, 34)	41	94	0.89	10 (7, 17)	32	64	0.12
	**Male**	18 (12, 25)	53			19 (9, 46)	32		

**Race**	**White**	17 (13, 26)	66	93	0.65	14 (8, 25)	43	64	0.59
	**NonWhite**	22 (8, 26)	27			10 (6, 19)	21		

**Grade**	**1/2**	20 (16, 26)	74	93	0.003	14 (10, 19)	52	64	0.031
	**3/4**	7 (6, 14)	19			6 (2, 25)	12		

**Lymph Nodes**	**0**	26 (22, 48)	27	92	0.009	21 (14, ∞)	20	63	0.008
	**≥ 1**	15 (12, 20)	65			9 (7, 12)	43		

**Tumor size by**	**1/2**	12 (6, 22)	15	93	0.45	19 (5, ∞)	9	64	0.31
**TNM stage**	**3/4**	18 (15, 26)	78			11 (8, 19)	55		

**Treatment**	**Chemo**	20 (15, 26)	82	94	<.001	14 (9, 19)	62	64	0.013
	**No chemo**	6 (3, ∞)	12			5	2		

**RRM1**	**Low**	20 (15, 25)	61	93	0.50	12 (8, 21)	40	63	0.76
**IHC Interp**.	**High**	14 (8, 48)	32			17 (5, ∞)	23		

**RRM1**	**Low**	24 (16, 26)	39	49	0.21	14 (8, 21)	28	34	0.48
**PCR Interp**.	**High**	48 (3, 48)	10			17 (11, 17)	6		

**ERCC**	**Low**	20 (12, 25)	41	94	0.83	14 (8, 25)	26	64	0.39
**IHC Interp**.	**High**	17 (12, 26)	53			12 (7, 19)	38		

**ERCC**	**Low**	16 (12, 25)	46	79	0.39	10 (7, 19)	29	56	0.74
**PCR Interp**.	**High**	22 (9, 48)	33			14 (6, 21)	27		

Abbreviations: ∞ = infinity; Interp. = Interpretation

**Table 4:** Multivariate cox regression analysis for overall survival

A. Survival in Pancreatic CA: Relative Gene Expression (N = 42)			
		**Multivariate**	
		
**Variable (Comparison)**		**Hazard Ratio** **(95% CI)**	***p*-value^†^**

Year of Diagnosis	one year increase	0.97 (0.69, 1.35)	0.836
Age	≥ 65.5 vs. < 65.5	1.53 (0.54, 4.33)	0.419
Positive Lymph Nodes	Any vs. None	0.80 (0.29, 2.22)	0.672
Treatment received	No Chemo vs. Chemo	13.09 (1.71,100.2)	0.013
ERCC1 Rel Gene Expression (PCR)	Low vs. High	0.86 (0.26, 2.81)	0.797
RRM1 Rel Gene Expression (PCR)	Low vs. High	17.58 (1.45,213.5)	0.024

**B. Survival in Pancreatic CA: Protein Expression by IHC Score (N = 91)**			

		**Multivariate**	
		
**Variable (Comparison)**		**Hazard Ratio** **(95% CI)**	***p*-value^†^**

Year of Diagnosis	one year increase	0.86 (0.71, 1.02)	0.088
Age	≥ 65.5 vs. < 65.5	1.36 (0.74, 2.52)	0.320
Positive Lymph Nodes	Any vs. None	2.34 (1.21, 4.52)	0.011
Treatment	No Chemo vs. Chemo	3.04 (1.25, 7.41)	0.014
ERCC1 IHC	Low vs. High	0.96 (0.53, 1.74)	0.888
RRM1 IHC	Low vs. High	0.83 (0.42, 1.63)	0.583

Stratified by *Grade*

† Cox regression

**Table 5:** Overall and Disease-Free Survival using Kaplan-Meier and log-rank test but only in patients who received complete chemotherapy regimen (all of whom received gemcitabine)

		Overall Survival				Disease-Free Survival			
		
Covariate		Median [months] (95% CI)	N	Total N	Log-rank *p*-value	Median [months] (95% CI)	N	Total N	Log-rank *p*-value
**Overall**		20.3 (15, 25.5)	82	82		13.5 (9.3, 19.4)	62	62	

**Age**	**Young (≤ 65.5)**	22 (15.6, 47.2)	44	82	0.18	10.5 (8, 19.3)	38	62	0.48
	**Old (> 65.5)**	17 (9.6, 25.5)	38			14 (8, ∞)	24		

**Size**	**Small (≤ 2.0)**	25 (9.6, 47.2)	18	82	0.45	20.5 (8.3, ∞)	12	62	0.21
	**Large (> 2.0)**	17.7 (14.8, 25.5)	64			12 (8, 17.3)	50		

**Gender**	**Female**	22 (14.8, 34.5)	36	82	0.85	10 (6.5, 17.3)	31	62	0.12
	**Male**	20 (14.4, 25.5)	46			19.3 (9.4, 46)	31		

**Race**	**White**	20 (14.8, 26.5)	59	82	0.88	13.5 (8, 25.5)	42	62	0.65
	**Non-White**	22 (9.2, 26.2)	23			10.3 (5.5, 592)	20		

**Grade**	**1/2**	20.5 (16, 25.5)	67	82	0.028	14 (10.3, 20.5)	51	62	0.0503
	**3/4**	14.5 (6.5, 26.5)	15			9.4 (2, 25.5)	11		

**Lymph Nodes**	**0**	25.5 (15, 48.5)	26	81	0.038	20.5 (13.5, ∞)	20	61	0.012
	**≥ 1**	16 (13, 20.3)	55			9.4 (6.5, 14)	41		

**Tumor**	**1/2**	20 (6.5, 47)	13	82	0.59	19.5 (5.5, ∞)	8	62	0.21
**(TNM)**	**3/4**	20.5 (15, 26)	69			12 (8, 19.3)	54		

**RRM1**	**Low**	20.3 (15, 25.5)	55	81	0.87	13.5 (8, 20.5)	39	61	0.84
**IHC Interp**	**High**	47.8 (8.7, 48)	26			16.6 (5.5, ∞)	22		

**RRM1**	**Low**	24 (17, 26)	37	44	0.070	14 (9.4, 25.5)	26	32	0.62
**PCR Interp**	**High**	48 (*, *)	7			17.3 (10.5, 17.3)	6		

**ERCC**	**Low**	20.3 (14.8, 26.5)	35	82	0.88	14 (9.4, 31.5)	25	62	0.36
**IHC Interp**	**High**	17 (13.3, 26)	47			12 (6.5, 20.5)	37		

**ERCC**	**Low**	17 (14.5, 24.7)	39	68	0.23	10 (7, 25.5)	28	54	0.74
**PCR Interp**	**High**	24 (11, 63.7)	29			13.5 (8, 20.5)	26		

* The 95% confidence interval could not be estimated since only one event (death) was recorded.

In regards to the correlation between IHC and PCR analysis, we found a borderline significant association between RRM1 expression by IHC and PCR (r = 0.26; *P *= 0.07) and the same result was applicable for ERCC1 expression (r = 0.21; *P *= 0.07). However there was a strong positive correlation between the RRM1 and the ERCC1 gene expression detection by PCR (r = 0.57; *P *< 0.0001) (Table 6).

**Table 6:** Correlation of IHC and PCR scores (Spearman Rank Correlation Coefficients)

	RRM1		ERCC1	
	
	RRM1 IHC	RRM1Relative Gene Exp	ERCC1 Relative Gene Exp	ERCC IHC
RRM1 IHC	1.00	0.26	0.13	0.18
		*P *= 0.07	*P *= 0.26	*P *= 0.09
	N = 93	N = 48	N = 75	N = 93

RRM1 Rel		1.00	0.57	0.06
Gene Exp			*P *< 0.0001	*P *= 0.66
		N = 49	N = 44	N = 49

ERCC1 Rel			1.00	0.21
Gene Exp				*P *= 0.07
			N = 76	N = 76

ERCC1 IHC				1.00
				N = 94

## Discussion

The results obtained in this study showed no significant correlation between the protein or mRNA expression levels of RRM1 and ERCC1 - detected by IHC or PCR - and OS or DFS in patients with resected PDA. Our data raise some questions regarding the real clinical and practical significance of analyzing these molecules as predictors of outcomes. Low levels of RRM1 did not predict better outcomes which, since the majority of our patients received gemcitabine based chemotherapy regimens, would have indirectly represented more tumor sensitivity to this agent.

The role of ERCC1 in PDA is less clear and since practically no patient received platinum analogue agents in this cohort, we cannot deduce the importance of ERCC1 as a predictor of oxaliplatin-based regimens as previously suggested [24,25]. Yet it seems to be clear that ERCC1 expression levels do not have any prognostic value in this patient cohort who did not receive platinum based chemotherapy. Future investigations should explore the significance of ERCC1 in patients receiving platinum based regimens.

Our results are discordant with what was previously reported by Akita and colleagues who found borderline better outcomes in patients with high expression levels of RRM1 and ERCC1 [20]. Nonetheless, in that study only a minority of patients received adjuvant chemotherapy (5/68) and in total only 40% received gemcitabine during the course of their treatment. Our population is unique and quite different to this previous work since the majority of our patients (87%) received gemcitabine in the adjuvant setting. Our study is more in accordance and relevant to current practices in the U.S. (i.e., standard of care therapy); hence our conclusions are directly applicable to the majority of patients who seek medical attention after a resection of a PDA. However the median OS in our cohort (18 months) was slightly inferior to that previously reported in randomized clinical trials (i.e.: in Conko-001, 22 months) [26]; this could be attributed to the more heterogeneous population of our study in comparison to one that could be observed in prospective and controlled clinical trials.

There are some studies that support the role of RRM1 over-expression as a source of resistance to gemcitabine in PDA [27,28]. One study showed that in gemcitabine resistant cell lines, sensitivity to this agent could be rescued by silencing the RRM1 expression (> 90%) with iRNA [10]. A similar correlation between RRM1 expression and gemcitabine efficacy was observed in the clinical setting but only in NSCLC [16]. However, during the last couple of years many other genes have been described as potential source of gemcitabine resistance in pancreatic tumoral cells, including human equilibrative nucleoside transporter-1 (hENT1) [29,30], deficiency in deoxycytidine kinase (dCK) [31], over-expression of RRM2 [32] and HuR [6,33]. The roles played by each particular gene in addition to the possible still undiscovered genes and pathways in the gemcitabine metabolism process, coupled with the complex in-vivo environment, are still under investigation [34]. However, some studies have already shown that the combination of detecting the expression of a select set of genes rather than the particular expression of one gene may play a more relevant, realistic clinical role [35,36]. Moreover, our own group has previously shown a notoriously more predictive value of HuR for gemcitabine sensitivity, which is most likely due to its ability to affect a myriad of downstream target genes [33]. Additionally, a recent report found also no prognosis value of RRM1 in PDA [37]

Taking all this evidence together, in conjunction with the results of the present study, we believe that the isolated detection of RRM1 or ERCC1 expression in PDA has little clinical relevance and deserves further investigation before formal recommendation regarding its use in clinical practice can be made. In addition, our study also serves as evidence that the information gathered from other tumor types (i.e.: lung cancer) should not always be directly applied to other cancers (especially to PDA where the aggressive biology appears to be unique) without the appropriate confirmation of these assumptions. In the particular cases of NSCLC, this discordance could also be partially explained by the fact that the overall response rate of gemcitabine in PDA is 5-7% [4] in comparison to the 20% found in NSCLC [38], meaning that lung cancer is intrinsically more sensitive to gemcitabine and levels of RRM1 may play a more relevant role.

Lastly, we should also take into consideration the fact that our study has some limitations which are mainly derived from its retrospective nature. We were not able to obtain reliable information to calculate DFS in one third of the cases since some patients were lost in follow up or the information available was inaccurate. As expected, this raises some concerns regarding selection bias in our cohort and conclusions regarding this matter should be analyzed cautiously and confirmed in future studies. However, we were able to estimate OS in all cases and, as mentioned before, given the very modest effect of second and third line chemotherapy regimens in PDA it is very unlikely that gross and undetected misbalance of these variables would have changed the results considerably. Additionally, we cannot directly translate our results to the metastatic setting since only 2 patients debuted with a stage IV disease. Other sources of variability could be related to the cut off values used for PCR detection or to the fact that only half of the patients studied had reliable information in regards to the gene expression, thus underscoring the difficulty of realistically utilizing this assay for clinical purposes. Indeed, when analyzing only those patients who received a complete regimen of chemotherapy, high RRM1 gene expression (PCR) showed a trend toward significance (*P *= 0.07) but the number of patients investigated was too small (N = 7) and results could be due to random sampling variability. We tried to correlate the gene and protein expression levels and we found a borderline significant correlation, but not a direct one. We cannot then discard the possibility that newer and more reliable techniques of gene expression detection could produce different results. Nonetheless, the current available evidence argues against this possibility.

## Conclusions

In conclusion, RRM1 and ERCC1 expression does not seem to have a clear predictive or prognostic value in resected PDA patients. Future studies will elucidate the role of these biomarkers in other tumor types and in PDA patients who present with metastatic disease.

## Competing interests

The authors declare that they have no competing interests.

## Authors' contributions

MEV participated in the conception and design of the study, collection and assembling of data, data interpretation and drafted the manuscript. TH carried out IHC and molecularstudies. BEL and EP performed the statistical analyses. SJL participated in the conception and design of the study, data interpretation and drafted the manuscript. CJY participated in the conception and design of the study. JRB participated in the conception and design of the study, data interpretation and drafted the manuscript. AKW participated in the conception and design of the study, data interpretation, carried out IHC and molecular studies, and drafted the manuscript. All authors read and approved the final manuscript.

## Pre-publication history

The pre-publication history for this paper can be accessed here:

http://www.biomedcentral.com/1471-2407/12/104/prepub
